# E-Cigarette Flavors and Frequency of E-Cigarette Use among Adult Dual Users Who Attempt to Quit Cigarette Smoking in the United States: Longitudinal Findings from the PATH Study 2015/16–2016/17

**DOI:** 10.3390/ijerph18084373

**Published:** 2021-04-20

**Authors:** Karin A. Kasza, Maciej L. Goniewicz, Kathryn C. Edwards, Michael D. Sawdey, Marushka L. Silveira, Shannon Gravely, Izabella Zandberg, Lisa D. Gardner, Geoffrey T. Fong, Andrew Hyland

**Affiliations:** 1 Department of Health Behavior, Roswell Park Comprehensive Cancer Center, Buffalo, NY 14263, USA; Maciej.Goniewicz@RoswellPark.org (M.L.G.); Andrew.Hyland@RoswellPark.org (A.H.); 2 Behavioral Health & Health Policy Practice, Westat Inc., Rockville, MD 20850, USA; KatyEdwards@westat.com; 3 Office of Science, Center for Tobacco Products, US Food and Drug Administration, Silver Spring, MD 20993, USA; Michael.Sawdey@fda.hhs.gov (M.D.S.); Izabella.Zandberg@fda.hhs.gov (I.Z.); Lisa.Wasson@fda.hhs.gov (L.D.G.); 4 National Institute on Drug Abuse, National Institutes of Health, Bethesda, MD 20852, USA; marushka.silveira@nih.gov; 5 Kelly Government Solutions, Rockville, MD 20850, USA; 6 Department of Psychology, University of Waterloo, Waterloo, ON N2L 3G1, Canada; shannon.gravely@uwaterloo.ca (S.G.); geoffrey.fong@uwaterloo.ca (G.T.F.); 7 School of Public Health and Health Systems, University of Waterloo, Waterloo, ON N2L 3G1, Canada; 8 Ontario Institute for Cancer Research, Toronto, ON M5G 0A3, Canada

**Keywords:** e-cigarette flavors, cigarette quit attempt, use frequency, dual use, population, longitudinal, US nationally representative, adults

## Abstract

Potential mechanisms by which e-cigarette use may relate to combustible cigarette smoking cessation are not well-understood. We used U.S. nationally representative data to prospectively evaluate the relationship between e-cigarette flavor use and frequency of e-cigarette use among adult cigarette/e-cigarette dual users who attempted to quit smoking cigarettes. Analyses used Population Assessment of Tobacco and Health (PATH) Study data from adult dual users (2015/16) who attempted to quit smoking between 2015/16 and 2016/17 (Wave 3-Wave 4, *n* = 685, including those who did/did not quit by 2016/17). E-cigarette flavor use (usual/last flavor, past 30-day flavor; assessed in 2015/16) was categorized into Only tobacco; Only menthol/mint; Only non-tobacco, non-menthol/mint; and Any combination of tobacco, menthol/mint, other flavor(s). The key outcome, evaluated at follow-up in 2016/17, was frequent e-cigarette use, which was defined as use on 20+ of past 30 days. Logistic regression was used to evaluate associations between e-cigarette flavor use in 2015/16 and frequent e-cigarette use at follow-up in 2016/17. Dual users who attempted to quit smoking had greater odds of frequent e-cigarette use at follow-up when they used only non-tobacco, non-menthol/mint flavor than when they used only tobacco flavor as their regular/last e-cigarette flavor (OR = 1.9, 95% CI: 1.1–3.4); findings were no longer significant when adjusted for factors including e-cigarette device type (AOR = 1.4, 95% CI: 0.7–2.8). Past 30-day e-cigarette flavor use results were generally similar, although frequent e-cigarette use at follow-up was highest among those who used any combination of tobacco, menthol/mint, or other flavors. Findings indicate that e-cigarette flavor use among dual users who attempt to quit smoking may be related to e-cigarette use frequency overall, which may indicate a mechanism underlying findings for e-cigarette use and smoking cessation. Further longitudinal research may help to disentangle how e-cigarette characteristics uniquely impact e-cigarette use frequency and smoking cessation/sustained use.

## 1. Introduction

A 2020 Cochrane Systematic Review concluded with moderate certainty that use of electronic nicotine products (“e-cigarettes”) increases combustible cigarette quit rates [1]; however, the role that various e-cigarette characteristics may play in combustible cigarette cessation behaviors remains unclear [2], and investigation into intermediate measures may provide insight on potential mechanisms of action underlying overall clinical trial findings. Analogous to nicotine gum manufacturers adding mint and orange flavors to their products to increase treatment adherence [3], so too may flavored e-cigarettes be more palatable to users, which could lead to greater use when trying to quit cigarette smoking, which could in turn impact the helpfulness of e-cigarette use for cigarette smoking cessation. Indeed, a randomized controlled trial in the United Kingdom among cigarette quit attempters found e-cigarette use to be more beneficial for quitting compared to nicotine replacement therapy use, with greater treatment adherence in the e-cigarette treatment arm compared to the nicotine replacement therapy treatment arm, and with fruit-flavored e-cigarettes most popular among participants [4]. However, these findings may not reflect what happens in the real world or among the U.S. population, and no e-cigarettes have been approved by the FDA as a cessation aid in the U.S. 

At the population level in the U.S., e-cigarette flavor use has been found to be associated with frequency of e-cigarette use in general [5], as has e-cigarette device type use, with users of open systems (i.e., refillable tanks) generally using more frequently than users of closed systems (i.e., disposables, devices with cartridges), and with users of open systems more likely to use non-tobacco flavors (e.g., fruit, candy) [6]. Greater frequency of e-cigarette use has been found to be associated with greater cigarette cessation in general [7,8,9], and Friedman and Xu found an overall positive relationship between use of non-tobacco flavor e-cigarettes (compared to use of tobacco-flavor e-cigarettes) and smoking cessation among adult cigarette smokers, although e-cigarette device type was not examined, and neither intentions to quit nor attempts to quit cigarette smoking were considered [10]. Kasza et al. found that e-cigarette flavor use was not associated with making a subsequent attempt to quit cigarette smoking [11], suggesting that the overall association found by Friedman and Xu may be driven by those making a quit attempt. 

We hypothesize that a possible mechanism of action behind findings from studies on e-cigarette use for smoking cessation may be in the association between e-cigarette flavor use and subsequent e-cigarette use frequency among smokers who attempt to quit. The aim of this study was to prospectively evaluate the association between e-cigarette flavor use and frequency of e-cigarette use one year later among adult cigarette/e-cigarette dual users who attempted to quit smoking cigarettes, while also considering possible confounding by e-cigarette device type, using the Population Assessment of Tobacco and Health (PATH) Study.

## 2. Materials and Methods

### 2.1. Participants 

The PATH Study is an ongoing, nationally representative, longitudinal cohort study in the U.S. Data were collected from October 2015 through October 2016 (Wave 3, referred to below as 2015/16) and from December 2016 through January 2018 (Wave 4, referred to below as 2016/17) using audio computer-assisted self-interviews administered in English or Spanish. The PATH Study was conducted by Westat and approved by the Westat Institutional Review Board. All respondents ages 18 and older provided informed consent. Details regarding the PATH Study design and methods [12], demographic and tobacco use distributions [13], and biomarkers of exposure related to tobacco product use [14] are published elsewhere. Details on interviewing procedures, questionnaires, sampling, weighting, response rates, and accessing the data are available at https://doi.org/10.3886/Series606 (accessed on 24 March 2021) [15]. 

Analyses in this paper included adults who smoked cigarettes and used e-cigarettes in the past 30 days (“dual users,” 62% of whom were daily cigarette smokers (95%CI: 58–66%) in 2015/16 and attempted to quit cigarette smoking between 2015/16 and 2016/17 (*n* = 685 for main analyses; average inter-wave duration across participants was one year), weighted to represent adults in the civilian, noninstitutionalized population in the U.S. through the application of longitudinal population and replicate weights that adjust for complex study design characteristics (e.g., oversampling) and nonresponse. 

### 2.2. Measures 

Dual use, frequency of cigarette smoking, frequency of e-cigarette use, regular/last e-cigarette flavor use, past 30-day e-cigarette flavor use, and e-cigarette device type use were assessed in 2015/16 and defined as described in Table 1. Attempting to quit cigarette smoking and frequency of e-cigarette use were assessed at follow-up in 2016/17 and defined as described in Table 1. Additionally, demographic characteristics were assessed in 2015/16 and categorized as follows: age group: 18–24 years, 25–39 years, 40–54 years, 55+ years; race/ethnicity: non-Hispanic White, non-Hispanic Black, Hispanic, other (including not reported); sex: male, female; sexual orientation: heterosexual/straight, not heterosexual/straight (including not reported).

### 2.3. Statistical Analyses 

Among dual users in 2015/16 who attempted to quit cigarette smoking between 2015/16 and 2016/17 (which includes those who had and had not quit in 2016/17), prevalence of frequent e-cigarette use (i.e., use on 20+ of past 30 days) at follow-up in 2016/17 was determined by regular/last e-cigarette flavor use and e-cigarette device type use in 2015/16. Logistic regression was used to evaluate associations between regular/last e-cigarette flavor use and frequent e-cigarette use at follow-up, and between e-cigarette device type use and frequent e-cigarette use at follow-up, and the interaction between regular/last e-cigarette flavor use and e-cigarette device type use was tested. Results are presented unadjusted and adjusted for the following measures, which were all assessed in 2015/16: age category, race/ethnicity, sex, sexual orientation, frequency of cigarette smoking, regular/last e-cigarette flavor use (in analyses assessing association for e-cigarette device type), frequency of e-cigarette use, and e-cigarette device type use (in analyses assessing associations for e-cigarette flavor use); see also Table 2 footnotes. Additionally, a set of parallel analyses was conducted for the past 30-day e-cigarette flavor use measure among dual users who used their primary e-cigarette product on one or more of the past 30 days in 2015/16 and were asked about past 30-day flavor use (*n* = 516). All analyses were weighted to produce nationally representative estimates, and 95% confidence intervals (CI) were computed using the balanced repeated replication method [16] with Fay’s adjustment set to 0.3 to increase estimate stability [17]. Analyses were conducted using Stata 15 software (StataCorp LLC, College Station, TX, USA) [18]. 

## 3. Results

### 3.1. Regular/Last E-Cigarette Flavor Use and E-Cigarette Device Type Use in 2015/16 and Frequent E-Cigarette Use at Follow-Up in 2016/17

Among dual users in 2015/16 who attempted to quit cigarette smoking between 2015/16 and 2016/17, 25.5% (95% CI: 22.0–29.4) were frequent e-cigarette users at follow-up in 2016/17 (Table 2). Frequent e-cigarette use at follow-up was highest among those who used only non-tobacco, non-menthol/mint flavor(s) (31.3%, 95% CI: 26.5–36.6), followed by those who used any combination of tobacco, menthol/mint, or other flavor(s) (23.8%, 95% CI: 13.0–39.5), those who used only tobacco flavor (19.0%, 95% CI: 12.5–27.7), and those who used only menthol/mint flavor (17.4%, 95% CI: 11.3–25.9) as their regular/last flavor, yielding approximately two-fold higher odds of frequent e-cigarette use at follow-up for those who used only non-tobacco, non-menthol/mint flavor compared to those who used only tobacco flavor in the unadjusted analysis (odds ratio (OR) = 1.9, 95% CI: 1.1–3.4). However, this association became attenuated and nonsignificant after adjusting for age, race/ethnicity, sex, sexual orientation, frequency of cigarette smoking, frequency of e-cigarette use, and e-cigarette device type use (adjusted odds ratio (AOR) = 1.4, 95% CI: 0.7–2.8). 

Those who used open system e-cigarette device types had greater odds of frequent e-cigarette use at follow-up (OR = 2.3, 95% CI: 1.4–4.0), and those who used disposable e-cigarette device types had lower odds of frequent e-cigarette use at follow-up (OR = 0.5, 95% CI: 0.2–1.0), each compared to those who used cartridge device types in the unadjusted model, but these associations became attenuated and nonsignificant after adjusting for other measures (AOR = 1.5, 95% CI: 0.8–2.8; AOR = 0.7, 95% CI: 0.3–1.6; respectively, Table 2). The interaction between regular/last e-cigarette flavor use and e-cigarette device type was not statistically testable due to small sample sizes. 

### 3.2. Past 30-Day E-Cigarette Flavor Use and E-Cigarette Device Type Use in 2015/16 and Frequent E-Cigarette Use at Follow-Up in 2016/17

Among dual users who used their primary e-cigarette product on ≥1 days in the past 30 days in 2015/16 and attempted to quit cigarette smoking between 2015/16 and 2016/17 (*n* = 516), 29.3% (95% CI: 25.0–34.0) were frequent e-cigarette users at follow-up in 2016/17 (Table 2). Frequent e-cigarette use at follow-up was highest among those who used any combination of tobacco, menthol/mint, or other flavors (40.5%, 95% CI: 27.2–55.3), followed by those who used only non-tobacco, non-menthol/mint flavor(s) (31.1%, 95% CI: 25.9–36.8), those who used only menthol/mint flavor (23.6%, 95% CI: 14.6–35.8), and those who used only tobacco flavor (19.0%, 95% CI: 10.9–31.2) in the past 30 days, yielding a nearly three-fold higher odds of frequent e-cigarette use at follow-up for those who used any combination of flavors, and a nearly two-fold higher odds of frequent e-cigarette use at follow-up for those who used only non-tobacco, non-menthol/mint flavor(s), each compared to those who used only tobacco flavor in the unadjusted analysis (OR = 2.9, 95% CI: 1.2–7.1; OR = 1.9, 95% CI: 0.9–4.0; respectively, though the latter was not statistically significant). These associations became attenuated and nonsignificant after adjusting for other measures (AOR = 1.9, 95% CI: 0.7–5.1; AOR = 1.3, 95% CI: 0.5–3.2; respectively). 

Those who used open system e-cigarette device types had greater odds of frequent e-cigarette use at follow-up (OR = 2.5, 95% CI: 1.3–4.8), and those who used disposable e-cigarette device types had lower odds of frequent e-cigarette use at follow-up (OR = 0.3, 95% CI: 0.1–0.9), each compared to those who used cartridge device types in the unadjusted model, but these associations became attenuated and nonsignificant after adjusting for other measures (AOR = 1.7, 95% CI: 0.8–3.5; AOR = 0.4, 95% CI: 0.1–1.5; respectively, Table 2). The interaction between past 30-day e-cigarette flavor use and e-cigarette device type use was not statistically testable due to small sample sizes.

## 4. Discussion

These U.S. nationally representative findings indicate that adult cigarette/e-cigarette dual users in 2015/16 who attempted to quit smoking cigarettes between 2015/16 and 2016/17 had higher odds of frequent e-cigarette use at follow-up if they used only non-tobacco, non-menthol/mint flavor as their regular/last e-cigarette flavor than if they used only tobacco flavor as their regular/last e-cigarette flavor in 2015/16. However, findings were not statistically significant when adjusted for other factors including e-cigarette device type, which was also a significant predictor of frequent e-cigarette use in unadjusted models, with open system users having higher odds of frequent use at follow-up than cartridge device type users, and with disposable device type users having lower odds of frequent use at follow-up than cartridge device type users. Results were similar when evaluating past 30-day e-cigarette flavor use though dual users who used a combination of flavors in the past 30 days had the highest rate of frequent e-cigarette use at follow-up. 

While our findings here point to overall prospective associations between some e-cigarette characteristics and e-cigarette use frequency among cigarette quit attempters, findings were attenuated after controlling for other variables including baseline e-cigarette use frequency and baseline cigarette smoking frequency. Sample size precluded us from examining whether associations differed by frequency of e-cigarette use at baseline, but future research with larger samples can investigate whether our conditioning on baseline e-cigarette use frequency attenuated the true association, and larger samples can also be used to disentangle associations among e-cigarette characteristics themselves and to investigate downstream impacts on quitting cigarettes/sustaining dual use. 

Study limitations also included not assessing e-cigarette use *during* the cigarette quit attempt, not evaluating whether flavors relate to intentions to quit, and using data that were collected just as newer generation e-cigarette products using salt-based e-liquid formulations were beginning to enter the U.S. marketplace in 2015/16 (i.e., prior to JUUL or Puff Bar brands becoming popular in the U.S.). Nonetheless, initial findings here underscore an area of research to consider when evaluating the population-level impact of flavored e-cigarettes, with the majority of adult dual users in the U.S. who attempt to quit smoking cigarettes using only non-tobacco, non-menthol/mint flavor e-cigarettes, and the majority using open system devices, which were a combination of characteristics that were excluded from FDA’s enforcement priorities for e-cigarettes at the time this article was submitted [19].

## 5. Conclusions

The majority of adult dual users in the U.S. who attempted to quit smoking cigarettes between 2015/16 and 2016/17 used only non-tobacco, non-menthol/mint flavor e-cigarettes, and open system devices. Findings further indicate overall associations between these characteristics and frequency of e-cigarette use at follow-up among cigarette quit attempters in unadjusted analyses but not in adjusted analyses; further longitudinal research may help to disentangle how e-cigarette characteristics uniquely impact e-cigarette use frequency and smoking cessation/sustained use. Nonetheless, this study adds large-scale population-based data to the literature that begins to address a possible mechanism of action behind other studies’ conclusions about the use of e-cigarettes to help with smoking cessation.

## Figures and Tables

**Table 1:** Measures.

Measures in 2015/16	Categorizations	Questions Used in Categorizations
Dual users	(1) Those who smoked 100 or more cigarettes in their entire life and smoked a cigarette and used any electronic nicotine product (hereafter referred to as e-cigarettes) in the past 30 days	Respondents were asked: “In the past 30 days, have you smoked a cigarette, even one or two puffs?” “How many cigarettes have you smoked in your entire life? A pack usually has 20 cigarettes in it” and “In the past 30 days, have you used an electronic nicotine product, even one or two times? (Electronic nicotine products include e-cigarettes, vape pens, personal vaporizers and mods, e-cigars, e-pipes, e-hookahs and hookah pens).”
Frequency of cigarette smoking	(1) Nondaily smoking (2) Daily smoking	Dual users were asked: “Do you now smoke cigarettes every day/some days/not at all?”
Frequency of e-cigarette use	(1) Use on less than 20 days in the past 30 days (2) Use on 20+ days in the past 30 days (including daily use)	Dual users were asked: “Do you now use [e-cigarettes] every day/some days/not at all?” and those who did not use every day were asked: “On how many of the past 30 days did you use [primary e-cigarette product]?”
Regular/last e-cigarette flavor use	(1) Only tobacco flavor (2) Only menthol/mint flavor (3) Only non-tobacco, non-menthol/mint flavor (includes multiple flavors) * (4) Any combination of tobacco, menthol/mint, other flavor(s)	Dual users were asked: “What flavor is [your regular brand/the brand you last used]? Choose all that apply.” (Respondents were asked about flavor of regular brand if they had a regular brand; respondents were asked about flavor of brand last used if they did not have a regular brand) Response options were: “tobacco-flavored; menthol or mint; clove or spice; fruit; chocolate; an alcoholic drink (such as wine, cognac, margarita or other cocktails); a non-alcoholic drink (such as coffee, soda, energy drinks, or other beverages); candy, desserts, or other sweets; some other flavor.”
Past 30-day flavor use	(1) Only tobacco flavor (2) Only menthol/mint flavor (3) Only non-tobacco, non-menthol/mint flavor (includes multiple flavors) ** (4) Any combination of tobacco, menthol/mint, other flavor(s)	Dual users who reported using their primary e-cigarette product on one or more of the past 30 days were asked: “In the past 30 days, which flavors of [primary e-cigarette product] have you used? Choose all that apply.” Response options were the same as above for regular/last flavor use.
E-cigarette device type use	(1) Disposable (not rechargeable) (2) Cartridge (rechargeable, uses cartridges) (3) Open system (rechargeable, does not use cartridges, refillable)	Is your [e-cigarette] rechargeable?; Does your [e-cigarette] use cartridges?; Can you refill your [e-cigarette] with “e-liquid”?
**Measures in 2016/17**	**Categorizations**	**Questions Used in Categorizations**
Attempted to quit cigarette smoking	Attempted to quit: Answered yes to having tried to quit completely in the past 12 months or is currently smoking not at all	Dual users in 2015/16 were asked in 2016/17: “In the past 12 months, have you tried to quit [cigarettes/tobacco] *** completely?” and “Do you now smoke cigarettes every day/some days/not at all?”
Frequent e-cigarette use	Use on 20+ days in the past 30 days (including daily use) vs. use on less than 20 days in the past 30 days (including no use at all)	Dual users in 2015/16 were asked in 2016/17: “Do you now use [e-cigarettes] every day/some days/not at all?” and those who did not use every day were asked: “On how many of the past 30 days did you use [primary e-cigarette product]?”

* Distribution of regular/last flavor use in the ‘Only non-tobacco, non-menthol/mint flavor’ category (corresponding to left-side column in Table 2) is as follows (respondents may endorse multiple flavors, thus percentages do not sum to 100%): clove or spice (2%, 95% CI: 1–4); fruit (57%, 95% CI: 52–62); chocolate (4%, 95% CI: 2–7); an alcoholic drink (3%, 95% CI: 2–6); a non-alcoholic drink (6%, 95% CI: 4–9); candy, desserts, or other sweets (48%, 95% CI: 42–53); some other flavor (4%, 95% CI: 2–6). Among respondents who used ‘Only non-tobacco, non-menthol/mint flavor,’ 82% (95% CI: 78–85) used one flavor, 14% (95% CI: 10–17) used two flavors, and 5% (95% CI: 3–7) used three or more flavors. ** Distribution of past 30-day flavor use in the ‘Only non-tobacco, non-menthol/mint flavor’ category (corresponding to right-side column in Table 2) is as follows (respondents may endorse multiple flavors; thus, percentages do not sum to 100%): clove or spice (2%, 95% CI: 1–4); fruit (60%, 95% CI: 54–66); chocolate (6%, 95% CI: 3–10); an alcoholic drink (4%, 95% CI: 2–7); a non-alcoholic drink (9%, 95% CI: 6–12); candy, desserts, or other sweets (55%, 95% CI: 47–62); some other flavor (5%, 95% CI: 3–9). Among respondents who used ‘Only non-tobacco, non-menthol/mint flavor,’ 69% (95% CI: 62–75) used one flavor, 24% (95% CI: 19–30) used two flavors, and 7% (95% CI: 5–10) used three or more flavors. *** Respondents who were dual users in 2015/16 and reported smoking cigarettes "not at all" in 2016/17 were categorized as having attempted to quit cigarette smoking completely. Respondents who were dual users in 2015/16 and reported smoking cigarettes "every day" or "some days" in 2016/17 were asked: "In the past 12 months, have you tried to quit [cigarettes/tobacco] completely?" Respondents were asked in reference to trying to quit "tobacco" if they ever used one or more non-e-cigarette tobacco product “fairly regularly” and now use it/them every day/some days. Otherwise, they were asked in reference to “cigarettes.” Respondents were categorized as having attempted to quit cigarette smoking completely if they responded “yes” to having tried to quit cigarettes/tobacco completely.

**Table 2:** Frequent e-cigarette use in 2016/17 by e-cigarette flavor and e-cigarette device type use in 2015/16 among adult dual users who attempted to quit smoking between 2015/16 and 2016/17.

Measures	Frequent E-Cigarette Use in 2016/17 (20+ Days vs. <20 Days in Past 30 Days)					Measures	Frequent E-Cigarette Use in 2016/17 (20+ Days vs. <20 Days in Past 30 Days)				
	% [95% CI]	OR [95% CI]	*p*	AOR ^3^ [95% CI]	*p*		% [95% CI]	OR [95% CI]	*p*	AOR ^6^ [95% CI]	*p*
**All adult dual users ^1^** **(*n* = 685)**	25.5 [22.0, 29.4]					**Adult dual users who used primary electronic nicotine product on 1+ days in past 30 days ^4^ (*n* = 516)**	29.3 [25.0, 34.0]				
**Regular/last e-cigarette flavor use in 2015/16 ^2^**						**Past 30-day e-cigarette flavor use in 2015/16 ^5^**					
**Only tobacco flavor** *n* = 141, 24% (95% CI: 19–28) of users	19.0 [12.5, 27.7]	ref		ref		**Only tobacco flavor** *n* = 86, 20% (95% CI: 15-26) of users	19.0 [10.9, 31.2]	ref		ref	
**Only menthol/mint flavor** *n* = 102, 15% (95% CI: 12–18) of users	17.4 [11.3, 25.9]	0.9 [0.4, 1.8]	0.771	1.3 [0.5, 3.2]	0.560	**Only menthol/mint flavor** *n* = 64, 13% (95% CI: 10–16) of users	23.6 [14.6, 35.8]	1.3 [0.5, 3.3]	0.563	2.2 [0.8, 6.3]	0.150
**Only non-tobacco, non-menthol/mint flavor(s)** *n* = 373, 51% (95% CI: 46–56) of users	31.3 [26.5, 36.6]	**1.9** **[1.1, 3.4]**	**0.021**	1.4 [0.7, 2.8]	0.342	**Only non-tobacco, non-menthol/mint flavor(s)** *n* = 281, 50% (95% CI: 44–56) of users	31.1 [25.9, 36.8]	1.9 [0.9, 4.0]	0.079	1.3 [0.5, 3.2]	0.553
**Any combination of tobacco, menthol/mint, or other flavor(s)** *n* = 69, 11% (95% CI: 8–14) of users	23.8 [13.0, 39.5]	1.3 [0.5, 3.4]	0.539	0.8 [0.3, 2.1]	0.630	**Any combination of tobacco, menthol/mint, or other flavor(s)** *n* = 85, 17% (95% CI: 13–21) of users	40.5 [27.2, 55.3]	**2.9** **[1.2, 7.1]**	**0.021**	1.9 [0.7, 5.1]	0.222
**E-cigarette device type use in 2015/16**						**E-cigarette device type use in 2015/16**					
**Cartridge** *n* = 197, 28% (95% CI: 25–32) of users	19.4 [12.9, 28.1]	ref		ref		**Cartridge** *n* = 151, 28% (95% CI: 24–32) of users	20.8 [13.1, 31.5]	ref		ref	
**Disposable** *n* = 128, 20% (95% CI: 16–24) of users	9.8 [5.8, 16.2]	**0.5** **[0.2, 1.0]**	**0.047**	0.7 [0.3, 1.6]	0.369	**Disposable** *n* = 71, 15% (95% CI: 12–20) of users	7.1 ^†^ [3.0, 16.0]	**0.3** **[0.1, 0.9]**	**0.033**	0.4 [0.1, 1.5]	0.176
**Open system** *n* = 342, 50% (95% CI: 45–54) of users	36.1 [31.0, 41.5]	**2.3** **[1.4, 4.0]**	**0.003**	1.5 [0.8, 2.8]	0.205	**Open system** *n* = 284, 55% (95% CI: 50–61) of users	39.9 [34.3, 45.8]	**2.5** **[1.3, 4.8]**	**0.005**	1.7 [0.8, 3.5]	0.158
**Unknown** *n* = 18, 2% (95% CI: 1–4) of users	7.8 ^†^ [1.0, 40.6]	0.4 [0.0, 5.2]	0.444	0.7 [0.0, 10.0]	0.772	**Unknown** *n* = 10, 1% (95% CI: 1–3) of users	23.9 ^†^ [5.4, 63.3]	1.2 [0.2, 9.6]	0.868	2.3 [0.3, 17.9]	0.415

Notes. %s, odds ratios (OR), adjusted odds ratios (AOR), and 95% Confidence Intervals (CI) are weighted; sample sizes are unweighted. Bold font indicates statistically significant association at alpha = 0.05. Average inter-survey duration between 2015/16 and 2016/17 was one year. For each flavor use variable, the interaction between flavor use and device type was not statistically significant at alpha=0.05 in unadjusted or adjusted models. ^†^ Estimate should be interpreted with caution; it has low statistical precision; it is based on a denominator sample size of less than 50, or the coefficient of variation of the estimate is larger than 30%. ^1^ Dual users were defined as those who smoked a cigarette and used an e-cigarette in the past 30 days. ^2^ Dual users who had used an e-cigarette in the past 30 days were asked: “What flavor is [your regular brand/the brand you last used] *? Choose all that apply.” Response options were: “tobacco-flavored; menthol or mint; clove or spice; fruit; chocolate; an alcoholic drink (such as wine, cognac, margarita or other cocktails); a non-alcoholic drink (such as coffee, soda, energy drinks, or other beverages); candy, desserts, or other sweets; some other flavor.” E-cigarette flavor(s) used were categorized into the following groups: (1) tobacco-flavor only, (2) menthol/mint-flavor only, (3) non-tobacco, non-menthol/mint flavor(s) only, and (4) any combination of tobacco, menthol/mint, other flavor(s). * Respondents were asked about flavor of regular brand if they had a regular brand; respondents were asked about flavor of brand last used if they did not have a regular brand. ^3^ Adjusted for age category, race/ethnicity, sex, sexual orientation, frequency of cigarette smoking, regular/last e-cigarette flavor use, frequency of e-cigarette use, and e-cigarette device type use, all assessed in 2015/16. ^4^ Dual users who smoked a cigarette and used their primary e-cigarette product on one or more of the past 30 days. ^5^ Dual users who reported using their primary e-cigarette product on one or more days in the past 30 days were asked: “In the past 30 days, which flavors of [primary e-cigarette product] have you used? Choose all that apply.” Response options were: “tobacco-flavored; menthol or mint; clove or spice; fruit; chocolate; an alcoholic drink (such as wine, cognac, margarita or other cocktails); a non-alcoholic drink (such as coffee, soda, energy drinks, or other beverages); candy, desserts, or other sweets; some other flavor.” E-cigarette flavor(s) used were categorized into the following groups: (1) tobacco-flavor only, (2) menthol/mint-flavor only, (3) non-tobacco, non-menthol/mint flavor(s) only, and (4) any combination of tobacco, menthol/mint, other flavor(s). ^6^ Adjusted for age category, race/ethnicity, sex, sexual orientation, frequency of cigarette smoking, past 30-day e-cigarette flavor use, frequency of e-cigarette use, and e-cigarette device type use, all assessed in 2015/16.

## Data Availability

Data may be obtained from a third party and are not publicly available (https://www.icpsr.umich.edu/icpsrweb/NAHDAP/studies/36231, accessed on 24 March 2021). Applications, instructions and conditions of use are available at the website above.

## References

[1] Hartmann-Boyce J., McRobbie H., Lindson N., Bullen C., Begh R., Theodoulou A., Hajek P. (2020). Electronic cigarettes for smoking cessation. Cochrane Database Syst. Rev..

[2] National Academies of Sciences Engineering and Medicine (2018). Public Health Consequences of e-Cigarettes.

[3] Rose J.E. (1996). Nicotine addiction and treatment. Annu. Rev. Med..

[4] Peter H., Anna P.W., Dunja P., Francesca P., Katie M.S., Natalie B., Qi W. (2019). A randomized trial of e-cigarettes versus nicotine-replacement therapy. N. Engl. J. Med..

[5] Chen J.C. (2018). Flavored E-cigarette Use and Cigarette Smoking Reduction and Cessation—A Large National Study among Young Adult Smokers. Subst. Use Misuse.

[6] Coleman B., Chang J.T., Rostron B.L., Johnson S.E., Das B., Valle-Pinero D., Arseima Y. (2019). An Examination of Device Types and Features Used by Adult Electronic Nicotine Delivery System (ENDS) Users in the PATH Study, 2015–2016. Int. J. Environ. Res. Public Health.

[7] Berry K.M., Reynolds L.M., Collins J.M., Siegel M.B., Fetterman J.L., Hamburg N.M., Stokes A. (2019). E-cigarette initiation and associated changes in smoking cessation and reduction: The Population Assessment of Tobacco and Health Study, 2013–2015. Tob. Control.

[8] Buu A., Hu Y.H., Piper M.E., Lin H.C. (2018). The association between e-cigarette use characteristics and combustible cigarette consumption and dependence symptoms: Results from a national longitudinal study. Addict. Behav..

[9] Coleman B., Rostron B., Johnson S.E., Persoskie A., Pearson J., Stanton C., Hyland A. (2018). Transitions in electronic cigarette use among adults in the Population Assessment of Tobacco and Health (PATH) Study, Waves 1 and 2 (2013–2015). Tob. Control.

[10] Friedman A.S., Xu S. (2020). Associations of Flavored e-Cigarette Uptake with Subsequent Smoking Initiation and Cessation. JAMA Netw. Open.

[11] Kasza K.A., Edwards K.C., Gravely S., Coleman B., Kimmel H., Everard C., Hyland A. (2021). Adults’ E-cigarette Flavor Use and Cigarette Quit Attempts, PATH Study Findings. Am. J. Prev. Med..

[12] Hyland A., Ambrose B.K., Conway K.P., Borek N., Lambert E., Carusi C., Compton W.M. (2017). Design and methods of the Population Assessment of Tobacco and Health (PATH) Study. Tob. Control.

[13] Kasza K.A., Ambrose B.K., Conway K.P., Borek N., Taylor K., Goniewicz M.L., Hyland A.J. (2017). Tobacco-product use by adults and youths in the United States in 2013 and 2014. N. Engl. J. Med..

[14] Goniewicz M.L., Smith D.M., Edwards K.C., Blount B.C., Caldwell K.L., Feng J., Hyland A.J. (2018). Comparison of Nicotine and Toxicant Exposure in Users of Electronic Cigarettes and Combustible Cigarettes. JAMA Netw. Open.

[15] Population Assessment of Tobacco and Health (PATH) Study.

[16] McCarthy P.J. (1969). Pseudoreplication: Further Evaluation and Applications of the Balanced Half-Sample Technique. Vital Health Stat Ser. 2.

[17] Judkins D.R. (1990). Fay’s method for variance estimation. J. Off. Stat..

[18] (2019). Stata Statistical Software.

[19] U.S. Department of Health and Human Services, Food and Drug Administration, Center for Tobacco Products (2020). Enforcement Priorities for Electronic Nicotine Delivery Systems (ENDS) and Other Deemed Products on the Market without Premarket Authorization.

